# Computed tomography calcium scoring association and reclassification of clinical cardiovascular risk in asymptomatic Mexican patients

**DOI:** 10.1177/2050312120938233

**Published:** 2020-07-02

**Authors:** Aldo Javier Vázquez Mézquita, Michelle Claire Williams, Rafael Choza Chenhalls, Nancy Berenice Guzmán Martínez, Ana Patricia Chischistz Condey, Maria José Acosta Falomir, Marco Antonio Téliz Meneses, María Nayeli Vázquez Sánchez

**Affiliations:** 1 University/BHF Centre for Cardiovascular Science, The University of Edinburgh, Edinburgh, UK; 2 Department of Radiology and Molecular Imaging, Centro Médico ABC (The American British Cowdray Medical Center), Mexico City, Mexico; 3 Department of Radiology, Biomédica de Referencia, Mexico City, Mexico

**Keywords:** ASCVD, cardiovascular, coronary calcium scoring, Framingham, Mexican, reclassification

## Abstract

**Objectives:**

To establish tailored preventive treatment, we studied the ability of coronary artery calcium scoring to reclassify patients with intermediate cardiovascular risk and its association with additional risk factors in our Mexican preventive care center.

**Materials and methods:**

In this retrospective cohort study, we analyzed 520 asymptomatic patients from a Mexican primary prevention population between 2014 and 2018. Coronary artery calcium scoring, laboratory results, and anthropometric measurements (abdominal circumference and body mass index) were assessed. The Framingham risk score and American Heart Association/American College of Cardiology (AHA/ACC) atherosclerotic cardiovascular disease risk algorithm were calculated. Correlations between coronary artery calcium scoring, anthropometric measurements, and clinical cardiovascular risk scores were assessed. We assessed the ability of coronary artery calcium scoring to reclassify patients recommended for statin therapy compared with the cardiovascular risk scores.

**Results:**

Patients had a mean age of 67.5 years (*SD* ± 9.8) and 294 subjects (56.5%) were male. Coronary artery calcium scoring has a positive correlation with age, AHA/ACC atherosclerotic cardiovascular disease risk algorithm, and Framingham risk score (*p* < 0.001 for all). Coronary artery calcium scoring was prevalent, occurring in 63.2% of patients with a median Agatston score of 22 with and interquartile range of 178. Male gender, older age, smoking habit, diabetes, and abdominal circumference were independent predictors of coronary artery calcium scoring (*p* < 0.001). Coronary artery calcium scoring downwardly reclassified 44.9% of patients in intermediate cardiovascular risk categories by the AHA/ACC atherosclerotic cardiovascular disease risk algorithm and 43.9% by the Framingham risk score. Coronary artery calcium scoring upwardly reclassified 46.8% of patients in intermediate risk categories by the AHA/ACC atherosclerotic cardiovascular disease risk algorithm and 56% by the Framingham risk score.

**Conclusion:**

Coronary artery calcium scoring is prevalent in this Mexican primary prevention cohort and has the ability to reclassify a significant percentage of intermediate cardiovascular risk patients.

## Introduction

Coronary artery disease (CAD) remains a leading cause of death around the globe. Approximately, 17.5 million people died because of cardiovascular disease in 2012, which represented 30% of the total mortality worldwide, of which approximately, 7.4 million of these deaths were caused by CAD.^1^ Countries with low- and medium-income levels are the most affected in regions like Latin America, the Caribbean, Central and Eastern Europe, as well as Central Asia.^2^ In 2015, the death rate secondary to ischemic heart disease was higher in Central Latin America (119 per 100, 000) and Central Asia (336 per 100,000) compared with Western Europe (80 per 100, 000) and North America (106 per 100,000).^2,3^

To identify asymptomatic patients with CAD, we currently rely on clinical tools and cardiovascular risk scores, such as the Framingham risk score (FRS).^4^ Furthermore, they often include a limited diversity, including low numbers of women and ethnic or racial groups. Clinical parameters that may be used to assess cardiovascular risk include the assessment of visceral fat. However, limited information is available on how clinical assessment of visceral fat varies between populations of different ethnicities.^5,6^

Non-invasive imaging with computed tomography (CT) coronary artery calcium scoring (CACS) can be used to identify and quantify atherosclerotic plaque burden. This can be applied to identify CAD in patients who traditional risk scoring systems may miss, and who may benefit from preventive medication and lifestyle modification advice. This has important health economic implications.^7,8^ Previous studies have shown that CACS can reclassify patients compared with other scoring systems, such as the FRS^9^ or the atherosclerotic cardiovascular disease (ASCVD) risk algorithm by the American Heart Association (AHA) and the American College of Cardiology (ACC).^10–13^

In the Mexican population, a previous study by Posadas-Romero et al.,^14^ identified the prevalence of coronary artery calcified plaques in individuals between 34 and 75 years of age, 40% was for men and 14% for women. In the Multiethnic Study of Atherosclerosis (MESA), the prevalence of coronary artery calcified plaques in the Hispanic population was 57.5% in men and 35% in women.^14^ This overestimation of cardiovascular risk has been reported in other racial and ethnic groups.^11^

Even though, many studies that determine the association between cardiovascular risk and CACS have been performed in other countries, we currently do not have studies in Mexico that assess how many patients could be reclassified by CACS in other cardiovascular risk categories.

The purpose of detecting subjects at an intermediate or high cardiovascular risk is imperative for us to establish tailored preventive treatment, if necessary. We, therefore, analyzed a possible correlation between CACS, anthropometric measurements, and both cardiovascular risk scores (FRS and the AHA/ACC ASCVD risk algorithm) as a main objective. As a primary hypothesis, we considered that CACS has a positive correlation with anthropometric measurements and both cardiovascular risk scores.

## Methods and materials

### Study population

This was a single institution retrospective cohort study that included patients representing a middle- to high-income adult asymptomatic population in Mexico City and its surrounding metropolitan area. These are asymptomatic patients who self-refer to our preventive care unit in which CACS CT is offered to them. Ethical approval by the Research and Ethics Committee was obtained before the study began (ID number ABC 18-11); patients from our database were contacted and a written consent was obtained from them to use their data for this study. We included all asymptomatic male and female patients born in Mexico, who had imaging, clinical and laboratory information obtained on the same day in our institution. Exclusion criteria were the presence of previous vascular or cardiac interventional procedures and symptoms of CAD. Patients whose age or laboratory values could not be processed by the cardiovascular risk calculators or were incomplete (FRS and the AHA/ ACC ASCVD risk algorithm) were eliminated from the analysis.

### Assessment of cardiovascular risk factors

Data for each patient were obtained from their clinical records, including the age, gender, high-density lipoprotein (HDL) and low-density lipoprotein (LDL) levels, blood pressure levels, body mass index (BMI), abdominal circumference, smoking habit, diabetes mellitus, and systemic arterial hypertension, the latter previously diagnosed by their physicians.

### Assessment of cardiovascular risk scores

Clinical and laboratory data were used to calculate the FRS and AHA/ACC ASCVD score to determine each patient’s cardiovascular risk (%) for the next 10 years.^9–11^ Both calculators used age, gender, ethnicity, HDL and LDL levels, blood pressure level, diabetes mellitus, systemic hypertension, and smoking habit as parameters. Four radiology residents (A.J.V.-M, N.B.G-M, M.J.A.-F, and A.P.C-C) collected the information stored in each patient’s file and used it to calculate the cardiovascular risk scores with the aforementioned calculators.

Regarding the AHA/ACC ASCVD risk algorithm, statin therapy is not recommended in patients with a low-risk score (<5%), while in patients with an intermediate risk score (5%–7.5%), statins can be considered. In patients with an intermediate (7.5%–20%) or high-risk score (>20%), statin therapy is recommended.^12^

Regarding the FRS, statin therapy is not recommended in patients with a low cardiovascular risk (<10%). In contrast, statin therapy is recommended in patients with an intermediate (10%–20%) or high (>20%) cardiovascular risk.^9^

### Imaging technique and interpretation

Non-contrast electrocardiogram-gated CT of the heart was performed to assess CACS using a Philips Brilliance 64-slice CT scanner. Each slice has a 3 mm increment, tube voltage of 120 kVs, the effective dose was calculated by multiplying the dose length product (DLP) and a *w* conversion coefficient of 0.014. The approximate effective dose was 0.85 mSv. The official calcium scoring studies were interpreted by two radiologists specialized in cardiovascular imaging and two cardiologists specialized in cardiovascular imaging. The four specialists were not part of this protocol and were blinded from the clinical and laboratory data.

### Coronary artery calcium score

For the calcium scoring analysis, we used the Agatston score method^15^ to quantify coronary artery plaque calcification, which is based on detecting voxels with a value equal to or higher than 130 Hounsfield units. The result was classified using the Coronary Artery Calcium Data and Reporting System (CAC-DRS) categories published by the Society of Cardiovascular Computed Tomography:^16^ very low cardiovascular risk for a CACS of 0 (CAC-DRS 0), mildly increased cardiovascular risk for CACS between 1 and 99 (CAC-DRS 1), mildly to moderately increased cardiovascular risk for CACS between 100 and 299 (CAC-DRS 2), and moderately to severely increased cardiovascular risk for CACS equal to or higher than 300 (CAC-DRS 3). The score values used in this study were acquired from the official reports.

### Sample calculation

Our population was divided into gender (male or female). The average sensibility of CACS to detect calcified plaques is 90%. Regarding the prevalence of CAD in the Mexican population, a study by Posadas-Romero et al.^14^ indicated that men have a prevalence of around 40%, while women have a prevalence of 14%. Based on these data, the minimum number of male subjects required was 75, while the minimum number of female subjects needed was 215, considering an alpha value of 0.05, a power of 0.8. We decided to use the sample calculation based on the epidemiological data by Posadas-Romero et al.,^14^ which is explicitly based on the Mexican population.

### Statistical analysis and software processing

Statistical analysis was performed using SPSS Statistics (version 25 by IBM^®^). Normal distributed variables are presented in means and standard deviations, whereas, non-normally distributed variables are presented as medians and percentiles. The statistical significance was assessed using the t-test for independent variables, the Mann–Whitney U, chi-square, Kruskal–Wallis, Wilcoxon, Spearman’s rho, and multivariate regression tests as appropriate. A two-tailed *p*-value < 0.05 was considered statistically significant.

## Results

A total of 640 patients were found in our database, who were screened at our preventive care unit over 4 years (2014–2018). Of these, 579 consented to inclusion in this study. Forty-three were excluded because of chest pain symptoms at the time of assessment. A total of 16 patients were excluded because of previous cardiovascular procedures, such as coronary stents. A total of 520 asymptomatic patients with complete data were included, the majority were men with a total of 294 subjects (56.5%) with a mean age of 67.5 years (*SD* ± 9.8; Table 1). In the cardiovascular risk score stratification, 459 patients could be classified using the AHA/ACC ASCVD risk algorithm and 401 patients with the FRS.

**Table 1.** Demographic information.

		Overall	Male	Female	*p-value
Number of patients		520	294	226	
Age (years), mean ± *SD*		67.8 ± 9.5	67.5 ± 9.8	68 ± 9.2	0.089
Agatston calcium score, median (25th; 75th percentiles)		22 (0; 178)	50 (0; 267)	2 (0; 98.12)	<0.001
CAC-DRS, *n* (%)	CAC-DRS 0	191 (36.8)	83 (28.2)	108 (47.8)	<0.001
	CAC-DRS 1	152 (29.2)	89 (30.3)	63 (27.9)	
	CAC-DRS 2	83 (16)	51 (17.3)	32 (14.2)	
	CAC-DRS 3	94 (18)	71 (24.1)	23 (10.2)	
CACS > 0		329 (63.2)	211 (71.7)	118 (52.2)	
BMI mean ± *SD*		26.4 ± 4.3	26.6 ± 3.8	26.1 ± 4.8	0.158
Abdominal circumference (cm), mean ± *SD*		92 ± 11.8	96.5 ± 9.8	87 ± 12.1	<0.001
ACC/AHA ASCVD risk algorithm, median (25th; 75th percentiles)		16.6 (9; 27)	20.4 (12.6; 29.8)	13.4 (6.3; 22.4)	<0.001
FRS, median (25th; 75th percentiles)		19.8 (10.2; 31.9)	25 (16; 38.9)	12.7 (7.4; 21.6)	<0.001
Diabetes mellitus, *n* (%)		72 (13.8)	39 (13.3)	33 (14.6)	0.633
Systemic arterial hypertension, *n* (%)		197 (37.8%)	114 (38.8%)	83 (36.7%)	0.633
Smoking status, *n* (%)		214 (41.2%)	128 (43.5%)	86 (38.1%)	0.208

CAC-DRS: Coronary Artery Calcium Data and Reporting System; CACS: coronary artery calcium scoring; ACC: American College of Cardiology; AHA: American Heart Association; ASCVD: atherosclerotic cardiovascular disease; FRS: Framingham risk score; BMI: body mass index. *A *p*-value < 0.05 shows a statistically significant difference in the frequencies between the male and female groups.

**Figure 1.**
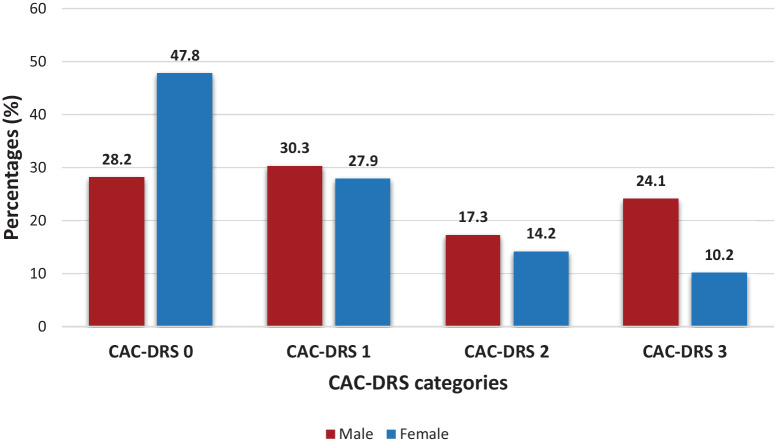
CAC-DRS frequencies determined by gender.

Coronary artery calcification was present in 63.2% (329) of patients with a median Agatston score of 22 (Table 1). The CAC-DRS category distribution by gender is illustrated in Figure 1.

### CACS correlation with cardiovascular risk scores and anthropometric measures

Overall, CACS and abdominal circumference indicated a low but positive correlation of 0.235 (*p* < 0.001). In the case of BMI, CACS had an overall low correlation coefficient of 0.134 (*p* < 0.01). Age and CACS indicated a correlation coefficient of 0.416 (*p* < 0.0001). Regarding cardiovascular risk scores, CACS and the AHA/ACC ASCVD risk algorithm had an overall correlation coefficient of 0.436 (*p* < 0.001). Meanwhile, CACS and the FRS had an overall positive correlation of 0.430 (*p* < 0.001). The correlation coefficient values for each gender are listed in Table 2.

**Table 2.** CACS correlation coefficients with anthropometric measures and cardiovascular risk scores.

	Overall	*p-value	Female	*p-value	Male	*p-value
Age	0.416	<0.001	0.43	<0.001	0.42	<0.001
Abdominal circumference	0.235	<0.001	0.284	<0.001	0.05	0.393
BMI	0.134	<0.01	0.204	<0.01	0.038	0.522
AHA/ACC ASCVD risk algorithm	0.436	<0.001	0.407	<0.001	0.36	<0.001
FRS	0.43	<0.001	0.379	<0.001	0.334	<0.001

AHA: American Heart Association; ACC: American College of Cardiology; ASCVD: atherosclerotic cardiovascular disease; CACS: coronary artery calcium scoring; FRS: Framingham risk score; BMI: body mass index.

* *p*-values < 0.05 were considered statistically significant.

### Coronary calcified plaque burden predictors

A multivariate regression analysis was performed, which determined that increased age, male gender, as well as diabetes mellitus and abdominal circumference are independent predictors for calcified plaque burden. However, BMI, systemic arterial hypertension, and a smoking habit were not independent risk predictors for calcified plaque burden. The results of the multivariate regression analysis are presented in Table 3.

**Table 3.** Multivariate regression analysis assessing predictors of CACS > 0. The *p*-values < 0.05 were considered statistically significant.

CAC > 0	*b*-value	Standard error	*p*-value	Odds ratio	95% confidence interval	
					Lower bound	Upper bound
Age	0.103	0.013	0.000	1.109	1.080	1.138
BMI	–0.058	0.047	0.215	0.944	0.861	1.034
Abdominal circumference	–0.278	0.277	0.025	1.042	1.005	1.081
Male gender	0.748	0.255	0.003	2.113	1.281	3.486
Smoking habit	0.170	0.217	0.434	1.185	0.774	1.814
Previous diagnosis of diabetes mellitus	0.875	0.358	0.014	2.400	1.191	4.837
Previous diagnosis of systemic arterial hypertension	0.134	0.224	0.551	1.143	0.736	1.775

CACS: coronary artery calcium scoring; CAC: coronary artery calcium; BMI: body mass index.

#### Comparison between CACS and AHA/ACC ASCVD risk categories

**Figure 2.**
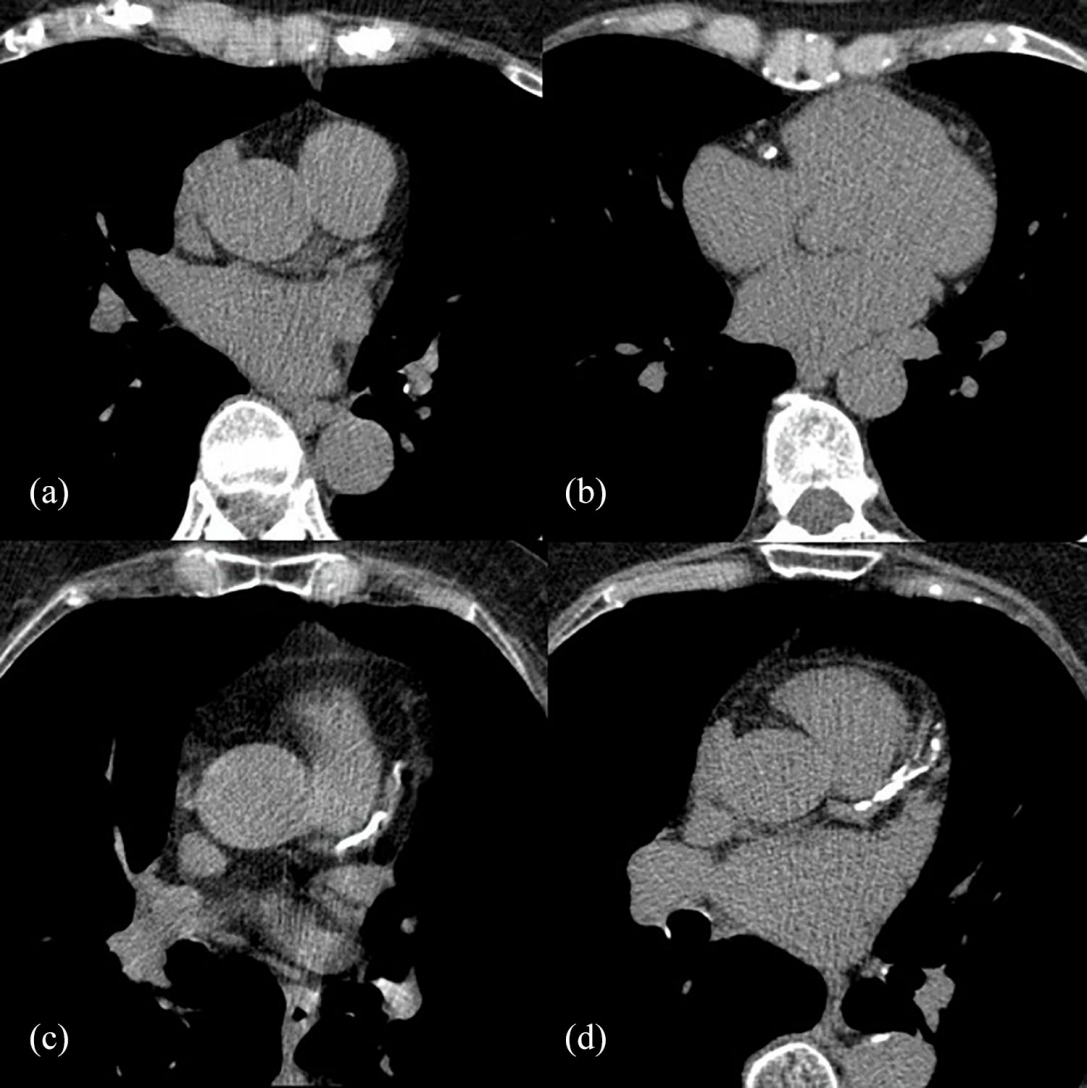
Patients classified by the AHA/ASCVD risk algorithm and CAC-DRS. (a) A 75-year-old female patient with a cardiovascular risk of 39% and CAC-DRS 0; in this case, statin drug therapy is recommended, regardless of CACS. (b) A 65-year-old female with a cardiovascular risk of 3.9% and CAC-DRS 1; upwardly reclassified with a statin drug therapy recommendation. (c) A 75-year-old female with a cardiovascular risk of 5.7% and CAC-DRS 3; upwardly reclassified with a statin drug therapy recommendation. (d) A 75-year-old male with a cardiovascular risk of 33.5% and CAC-DRS 3; this patient stayed in the high-risk category.

For the 459 subjects where the AHA/ACC ASCVD risk algorithm could be assessed, 65 (14.1%) patients were classified in the “statin not recommended” group, 207 (45%) patients in the “consider statin” group, and 187 (40.7%) in the “statin recommended” group. In contrast, with the influence of CACS, 158 (34.4%) patients were classified in the “statin not recommended” group and 284 (61.8%) in the “statin recommended” group. Only 17 (3.7%) patients stayed in the “consider statin” group. Overall, CACS reclassified patients in the intermediate cardiovascular risk categories, with 44.92% downward and 46.86% upward. The frequencies for each gender are listed in Table 4. There was a statistically significant difference between the distributions of the AHA/ACC ASCVD risk estimate with and without the CACS reclassification (*p* < 0.001), the values are listed in Table 5. In Figure 2, we present six examples of statin drug therapy recommendations determined by the AHA/ACC ASCVD risk algorithm and CACS.

**Table 4.** Statin recommendation based on CACS and cardiovascular risk based on the AHA/ACC ASCVD and FRSs.

		Overall (%)	*p-value* *	Female (%)	*p-value* *	Male (%)	*p-value* *
AHA/ACC ASCVD risk algorithm	Total sample *(n)*	459 (100)		203 (44.2)		256 (55.8)	
Statin not recommended	Without CACS	65 (14)	< 0.001	37 (57)	< 0.001	28 (43)	< 0.001
	CACS considered	158 (34)		100 (63.3)		58 (36.7)	
Consider statin	Without CACS	37 (8)	< 0.001	26 (70.3)	0.002	11 (29.7)	0.008
	CACS considered	17 (4)		10 (58.8)		7 (41.2)	
Statin recommended	Without CACS	357 (78)	< 0.001	140 (39.2)	< 0.001	217 (60.8)	< 0.001
	CACS considered	284 (62)		93 (32.7)		191 (67.3)	
FRS	Total sample *(n)*	401		178 (44.4)		223 (55.6)	
Statin not recommended	Without CACS	96 (24)	< 0.001	67 (69.8)	< 0.001	29 (30.2)	< 0.001
	CACS considered	143 (36)		99 (69.2)		44 (30.8)	
Statin recommended	Without CACS	305 (76)	< 0.001	111 (36.4)	< 0.001	194 (63.6)	< 0.001
	CACS considered	258 (64)		79 (30.6)		179 (69.4)	

CACS: coronary artery calcium scoring; AHA: American Heart Association; ACC: American College of Cardiology; ASCVD: atherosclerotic cardiovascular disease; FRS: Framingham risk score.

Values show number (%).

* *p*-values < 0.05 demonstrate a statistically significant difference in the frequencies for each statin recommendation with and without the influence of CACS.

**Table 5.** Statin recommendation based on CAC-DRS and ASCVD risk estimate.

Statin recommendation based on CAC-DRS and ASCVD risk estimate						Total
Gender	ASCVD risk estimate	CAC-DRS				
		Very low	Mildly increased	Moderately increased	Moderately to severely increased	
Female	< 5%	27	8	2	0	37
	5–7.5%	16 (–)	9	0	1	26
	7.5–20%	47 (–)	19 (+)	11 (+)	6 (+)	83
	> 20%	15	19	16	7	57
Male	< 5%	23	5	0	0	28
	5–7.5%	4 (–)	5	1	1	11
	7.5–20%	26 (–)	25 (+)	20 (+)	16 (+)	87
	> 20%	27	40	24	39	130
Total		185	130	74	70	459

Very low = CAC-DRS 0; mildly increased = CAC-DRS 1; moderately increased = CAC-DRS 2; moderately to severely increased = CAC-DRS 3. Blue = statin not recommended; yellow = consider for statin; orange = recommend statin. (–) = CAC can reclassify risk downwardly; (+) = CAC can reclassify risk upwardly. CAC-DRS: Coronary Artery Calcium Data and Reporting System; ASCVD: atherosclerotic cardiovascular disease. There was a statistically significant difference between the distributions of the AHA/ACC ASCVD risk estimate with and without the CACS reclassification (*p* < 0.001).

### Comparison between CACS and Framingham risk categories

Considering the whole sample of 401 subjects by the FRS without the influence of CACS, 96 (23.9%) were classified in the low-risk category, 107 (26.6%) in the intermediate risk category, and 198 (49.3%) in the high-risk category.

Regarding the FRS, 96 patients (23.9%) were included in the “statin not recommended” group and 305 patients (76.1%) were included in the “statin recommended” group. In contrast, with the influence of CACS, a total of 143 patients (35.66%) were included in the “statin not recommended” group, whereas 258 patients (64.4%) were included in the “statin recommended” group. Overall, CACS reclassified patients in the intermediate cardiovascular risk category, with 43.9% downward and 56.1% upward. The frequencies for each gender are listed in Table 4. There was a statistically significant difference between the groups with and without the influence of CACS (*p* < 0.001), the values are listed in Table 6.

**Table 6.** Statin recommendation based on CAC-DRS and Framingham risk estimate.

Statin recommendation based on CAC-DRS and Framingham risk estimate						Total
Gender	Framingham risk	CAC-DRS				
		Very low	Mildly increased	Moderately increased	Moderately to severely increased	
Female	Low risk (< 10%)	44	18	4	1	67
	Intermediate risk (10–20%)	32 (–)	13 (+)	10 (+)	3 (+)	58
	High risk (> 20%)	23	15	10	5	53
Male	Low risk (< 10%)	23	5	0	1	29
	Intermediate risk (10–20%)	15 (–)	20 (+)	7 (+)	7 (+)	49
	High risk (> 20%)	35	45	30	35	145
Total		172	116	61	52	401

Very low = CAC-DRS 0; mildly increased = CAC-DRS 1; moderately increased = CAC-DRS 2; moderately to severely increased = CAC-DRS 3.

Blue = statin not recommended; orange = recommend statin.

(-) = CAC can reclassify risk downwardly; (+) = CAC can reclassify risk upwardly. CAC-DRS: Coronary Artery Calcium Data and Reporting System. There was a statistically significant difference between the distributions of the Framingham risk score with and without the CACS reclassification (*p* < 0.001).

## Discussion

We have shown that coronary artery calcification is frequent in our primary prevention cohort and that its presence or absence can reclassify patients based on both the FRS and AHA/ACC ASCVD risk score. CACS reclassified most of the patients with intermediate cardiovascular risk into lower or higher categories of both the FRS and AHA/ACC ASCVD risk score. Older age, male gender, smoking habit, as well as a previous diagnosis of diabetes mellitus, and abdominal circumference were independent risk predictors of coronary calcified plaque burden.

Greenland et al.^12^ recommended CACS as a tool to reclassify cardiovascular risk compared with the AHA/ACC ASCVD risk algorithm.^17–19^ As an imaging tool that can be used to tailor patient treatment, CACS may reduce costs for hospitals and patients alike.^7,8^ Considering both cardiovascular risk scores, around 44% of patients with intermediate cardiovascular risk were reclassified downwards because of a CAC score of zero, which would indicate that they do not need statin therapy. In contrast, around 51% of patients in the intermediate risk category had a stronger recommendation to start statin therapy because of a CAC score > 0. The high number of reclassified patients in the intermediate group in our study could be due to the high proportion of male patients. Male patients had higher median CAC score compared with female patients, and thus were more frequently reclassified. Most of the patients with intermediate cardiovascular risk who were not reclassified were women and had a CACS less than 100. It is important to note that a CAC score of zero is correlated with a low mortality rate.^19^

In this study, we had a positive correlation between CACS and cardiovascular risk assessment tools (AHA/ACC ASCVD and Framingham). We were able to identify older age, male gender, as well as a previous diagnosis of diabetes mellitus and abdominal circumference as independent predictors for the presence of calcified coronary plaques, similar to previous studies.^5,6,9,19^ Smoking was more common in the male population than the female population. Nonetheless, both were significantly higher than the national prevalence of 8.7% in females and 27.1% in males reported in the last national Mexican addiction survey ENCODAT 2016–2017.^20^ The BMI of both male and female groups was similar (BMI = 26), which is slightly above the upper cut-off point for the “normal” category.^21^ In the case of abdominal circumference, the mean of both genders was above the upper limit of the “normal” category.^22^ The percentage of patients with diabetes mellitus (13–14%) is similar to the prevalence reported in the Mexican population.^23^ More than a third of the included patients had a previous diagnosis of systemic arterial hypertension, which is also similar to the prevalence reported in our country.^24^

Regarding CACS and BMI, we did find a positive correlation between these variables in the female population. Nonetheless, this was not the case in the male and overall groups. In our study, BMI was not identified as a predictor for calcified plaque burden. Oh et al.^6^ tried to identify a relationship between waist–height ratio and CACS progression over 4 years and found an increased calcified plaque burden in patients with high waist–height ratios. In our study, we measured abdominal circumference as a secondary marker of central obesity and determined that it is an independent risk predictor for calcified plaque burden, with a positive correlation in female patients; the latter was not the case in male patients. Although both studies measure different variables, both attempt to link central obesity and calcified coronary plaques.

In the study by Alashi et al.,^25^ 41% of asymptomatic patients in the Cleveland Clinic were reclassified using the ASCVD risk algorithm in lower or higher cardiovascular risk categories because of CACS. In comparison, our study was able to reclassify 190 patients, which corresponds to 36% of our sample. This result may suggest that Mexican patients form middle- to high-income groups cannot be equally reclassified as in developed countries, such as the United States, even though our subjects come from groups with an allegedly healthier lifestyle than the rest of the Mexican population.

It is important to mention that our study is valuable to our population, because we lacked information regarding how many patients in Mexico could be reclassified in their cardiovascular risk category by the influence of CACS. Normally, we base our clinical approaches in studies made in other populations, therefore, it is imperative to us to know how CACS could be used in a setting like ours.

### Limitations

Our study’s population does not represent the complete ethnic and economic groups found in the Mexican territory. Importantly, this population is part of the medium- and high-income classes of our society. Belonging to the low-income class has been considered a risk factor for CAD in the previous literature.^24^ In addition, downstream investigation, medication use, and subsequent outcomes are not available. We recommend future studies regarding the reclassification of cardiovascular risk by CACS in the low-income Mexican population, which may behave quite different from the population we studied. It is important to remember that cardiovascular risk by CACS may reduce unnecessary statin drug use therapy, which can become a constant and unbearable expense for people with low income. Moreover, it would avoid unnecessary drug side effects. Nonetheless, because of the smaller proportion of reclassified patients in our study, in comparison with the Alashi study in the United States, it is important to determine the proportion of patients of low-income population in Mexico that could be reclassified by the same method.

Importantly, it must be remembered that the absence of calcified coronary plaques does not exclude the presence of obstructive coronary artery stenoses or adverse plaque characteristics.^25^

In this study, we did not determine whether CACS is cost-effective as a preventive tool to establish a tailored statin therapy, as suggested by other literature.^7,8^ In our case, the annual medical checkups were paid for by the patients or their insurance companies. Therefore, we cannot establish the cost-effectiveness at a national healthcare level.

We currently do not know whether this reclassification allows for an increased accuracy in risk prediction, since no survival data are available. Therefore, we recommend a follow-up study over the next decade in order by measuring the event rate of coronary ischemic disease and myocardial infarction to determine the accuracy of this method.

## Conclusion

CACS is prevalent in our Mexican primary prevention population and reclassified the majority of patients in intermediate cardiovascular risk groups using both the AHA/ACC ASCVD and FRS. The reclassification by CACS offers a tailored preventive approach for asymptomatic patients, which could reduce healthcare costs and unnecessary drug side effects and identify patients who may be missed by risk scoring systems. Anthropometric measurements were only associated with calcified plaque burden in female patients.
